# A Reducing Milieu Renders Cofilin Insensitive to Phosphatidylinositol 4,5-Bisphosphate (PIP_2_) Inhibition*[Fn FN2]

**DOI:** 10.1074/jbc.M113.479766

**Published:** 2013-09-03

**Authors:** Bianca Schulte, Isabel John, Bernd Simon, Christoph Brockmann, Stefan A. Oelmeier, Beate Jahraus, Henning Kirchgessner, Selina Riplinger, Teresa Carlomagno, Guido H. Wabnitz, Yvonne Samstag

**Affiliations:** From the ‡ Institute for Immunology, Ruprecht Karls University, D-69120 Heidelberg, Germany,; the § Structural and Computational Biology Unit, European Molecular Biology Laboratory, D-69117 Heidelberg, Germany, and; the ¶ Institute of Process Engineering in Life Sciences, Section IV: Biomolecular Separation Engineering, Karlsruhe Institute of Technology (KIT), D-76131 Karlsruhe, Germany

**Keywords:** Cofilin, Oxidation-Reduction, Phosphatidylinositol Signaling, Redox Regulation, T Cell

## Abstract

Oxidative stress can lead to T cell hyporesponsiveness. A reducing micromilieu (*e.g.* provided by dendritic cells) can rescue T cells from such oxidant-induced dysfunction. However, the reducing effects on proteins leading to restored T cell activation remained unknown. One key molecule of T cell activation is the actin-remodeling protein cofilin, which is dephosphorylated on serine 3 upon T cell costimulation and has an essential role in formation of mature immune synapses between T cells and antigen-presenting cells. Cofilin is spatiotemporally regulated; at the plasma membrane, it can be inhibited by phosphatidylinositol 4,5-bisphosphate (PIP_2_). Here, we show by NMR spectroscopy that a reducing milieu led to structural changes in the cofilin molecule predominantly located on the protein surface. They overlapped with the PIP_2_- but not actin-binding sites. Accordingly, reduction of cofilin had no effect on F-actin binding and depolymerization and did not influence the cofilin phosphorylation state. However, it did prevent inhibition of cofilin activity through PIP_2_. Therefore, a reducing milieu may generate an additional pool of active cofilin at the plasma membrane. Consistently, in-flow microscopy revealed increased actin dynamics in the immune synapse of untransformed human T cells under reducing conditions. Altogether, we introduce a novel mechanism of redox regulation: reduction of the actin-remodeling protein cofilin renders it insensitive to PIP_2_ inhibition, resulting in enhanced actin dynamics.

## Teaser

**Background:** Cofilin is a key molecule for actin dynamics whose activity can be locally inhibited by PIP_2_.

**Results:** Changes in the cofilin structure upon reduction render cofilin insensitive to PIP_2_ inhibition.

**Conclusion:** Local effects of PIP_2_ on cofilin activity are determined by the redox microenvironment.

**Significance:** We discovered a mechanism of spatio-microenvironmental control of actin dynamics by cofilin reduction at the plasma membrane.

## Introduction

The actin-binding protein cofilin plays a central role in the costimulation of human T cells via its actin cytoskeleton-modulating activity (1, 2). It exerts its function both by severing actin filaments, yielding more free filament ends for polymerization, and by depolymerizing F-actin, providing actin monomers for the generation of new actin filaments. The overall effect of cofilin action on the cytoskeleton is one of increased actin dynamics and plasticity (for review, see Samstag *et al.* (3)). A well described cofilin regulatory mechanism is phosphorylation on serine 3, which leads to reversible cofilin inactivation and keeps cofilin in that state in resting T cells (4–6). During costimulation, cytosolic cofilin becomes activated by dephosphorylation (7) and executes its actin cytoskeleton-remodeling function, which is crucial for correct immune synapse formation (1, 8). In addition to phosphorylation, another negative regulator of cofilin activity is phosphatidylinositol 4,5-bisphosphate (PIP_2_),^4^ the binding of which leads to reversible inactivation of cofilin at the membrane (9). In tumor cells, PIP_2_ was shown to be involved in cell activation in two ways. 1) It binds actin-binding proteins, attaching the cytoskeleton to the membrane (10) and regulating the function of different actin-binding proteins; and 2) PIP_2_ is cleaved into the second messengers diacylglycerol and phosphatidylinositol 1,4,5-trisphosphate (11), whereby cofilin is released from PIP_2_ inhibition and contributes to actin dynamics (12). Recently, however, the role of PIP_2_ as a second messenger precursor after primary T cell activation was found to be even less important than its crucial effect on the actin cytoskeleton (13), allowing for the morphological changes downstream of costimulation.

Cofilin is also known to be redox-sensitive; upon oxidative stress, such as occurs locally during inflammation or in cancer, cofilin in T cells can get oxidized and thereby become inactive (14). This oxidation (at first making the T cells hyporesponsive to activation) can, as a long-term effect, even lead to cell death (15, 16). However, T cells can stay active in the face of oxidative stress at their primary site of action, *i.e.* the site of inflammation. This is achieved with the help of dendritic cells, which can provide a reducing intracellular milieu for nearby T cells by up-regulating thiols in the T cells. Thereby, they effectively rescue antigen-specific T cells from the negative effects of an oxidative milieu at the site of antigen encounter (17–19). By which exact mechanisms a more reducing milieu makes T cells less vulnerable to oxidative stress was so far unknown. Here, we show that the protein structure of cofilin is influenced by a reducing milieu, which prevents cofilin inactivation by PIP_2_, thus quantitatively enhancing its actin-remodeling activity in the cell.

## EXPERIMENTAL PROCEDURES

### 

#### 

##### Recombinant Cofilin Expression and Purification

The human cofilin gene (National Center for Biotechnology Information (NCBI) Gene ID 1072) was cloned into expression vector pETM11 (European Molecular Biology Laboratory (EMBL) Vector Collection) via NcoI and BamHI restriction sites. Single-point mutations were introduced into the construct with the QuikChange site-directed mutagenesis kit (Stratagene) to yield the cofilin C39A, C80A, C139A, and C147A mutants. Proteins were expressed as described previously (20) and purified via immobilized metal ion affinity chromatography on ProBond resin (Invitrogen). The wash buffers used were native wash buffer (20 mm Na_2_HPO_4_ (pH 6.3) and 500 mm NaCl) and imidazole wash buffer (20 or 50 mm imidazole in native wash buffer). Cofilin was eluted from the column with imidazole elution buffer (300 mm imidazole in native wash buffer), and the elution fraction buffer was immediately exchanged either against NMR buffer (10 mm sodium P_i_ (pH 6.8), 300 mm NaCl, 0.2 mm EGTA, and 1 mm NaN_3_) or 10 mm Tris-Cl (pH 8.0) on a PD-10 column. For NMR measurement, proteins were concentrated in a Vivaspin tube (Sartorius) to 10–14 mg/ml (protein concentration determined by *A*_280_ absorption measurement).

##### Mass Spectrometry

Recombinant WT cofilin was treated with 40 mm iodoacetamide (IAA) for 30 min and subjected to HPLC/electrospray ionization mass spectrometry for mass determination.

##### NMR Spectroscopy

WT cofilin was reduced or oxidized for 1.5 h on ice with 10 mm DTT and 100 μm or 10 mm H_2_O_2_, respectively. All spectra were acquired at 300 K with a Bruker 500 or 600 spectrometer (the latter equipped with a TXI CryoProbe). Processing and analysis of acquired spectra were done using the programs TopSpin (Bruker) and SPARKY Version 3.114 (21). Spectra were compared with the cofilin spectra recorded by Pope *et al.* (20), which were the basis of the Protein Data Bank (PDB) cofilin structure (ID 1Q8G; DTT-reduced cofilin). Chemical shift perturbations (CSPs) were calculated using the following formula: CSP (ppm) = √((Δδ_H_)^2^ + (0.2 × Δδ_N_)^2^). The amino acids that corresponded to peaks that exhibited CSPs > 0.1 ppm in a comparison between two spectra were marked on the PDB cofilin three-dimensional model using UCSF Chimera.

##### Molecular Dynamics Simulation

Simulations were performed using YASARA structure software package version 13 (22) and were run as detailed in the software manual. PDB structure 1Q8G was simulated with and without a disulfide bond between C39 and C80 in explicit water using the Amber03 force field (23). Total simulation time was 5000 ps. Average structures were calculated and compared in terms of root mean square deviations of individual atom positions.

##### Actin Depolymerization Assay

This assay was performed with recombinant proteins as described previously (14). Briefly, rabbit skeletal muscle actin (Cytoskeleton, Inc.) was resuspended in actin buffer (5 mm Tris-HCl (pH 8.0) and 0.2 mm CaCl_2_) at a concentration of 2 mg/ml and incubated on ice for 30 min to depolymerize any existing actin oligomers. Actin polymerization was initiated by adding 0.1 volume of actin polymerization buffer (500 mm KCl, 20 mm MgCl_2_, and 10 mm ATP) and carried out for 60 min at room temperature. For reduction of cofilin, 10 μg of protein was incubated with 10 mm DTT for 30 min at room temperature. For PIP_2_ treatment, a final concentration of 2 mm PIP_2_ was added to the cofilin sample, and the mixture was incubated for 30 min on ice. To analyze F-actin depolymerization by cofilin, 10 μg of untreated or reduced cofilin was combined with 20 μg of F-actin in a total volume of 150 μl of 10 mm Tris (pH 8.0) for 30 min at 30 °C. The samples were then centrifuged at 100,000 × *g* for 1 h at 20 °C. Supernatant and pellet were analyzed by 14% SDS-PAGE, followed by Coomassie Blue staining. Unprocessed images of the protein bands were quantified via densitometry with NIH ImageJ. WT cofilin and cofilin cysteine mutants (C39A, C80A, C139A, and C147A) were prepared as described above.

##### Human Peripheral Blood T Cell (PBT) Isolation and Cell Culture

Peripheral blood mononuclear cells were isolated from freshly drawn heparinized venous blood of healthy volunteers (obtained upon approval by the local ethics committee). Untouched T cells were purified from peripheral blood mononuclear cells with MACS® Pan T Cell Isolation Kit II (Miltenyi) as described previously (14) and, at 3 × 10^6^/ml, left to rest either for 1 h or overnight at 37 °C and 5% CO_2_ in RPMI 1640 medium and 10% (v/v) FCS. Raji B cells (ATCC CCL-86) were cultured at 37 °C and 5% CO_2_ in RPMI 1640 medium and 10% FCS.

##### Western Blot Analysis of Cofilin Cysteine Modifications in Human PBT Lysates

PBTs were lysed with 1% Nonidet P-40, and the intact nuclei were removed by centrifugation. The post-nuclear lysates were subjected to mock or reducing treatment with DTT for 30 min. All free thiols in the lysates were modified with excess methoxypolyethylene glycol maleimide (Mal-PEG; 10 mm, 5 kDa; Sigma-Aldrich). The extent of modifications was analyzed by Western blotting via detection of size changes in cofilin with an anti-total cofilin antiserum (produced in our laboratory).

##### Imaging Flow Cytometry of PBT-Antigen-presenting Cell (APC) Conjugates

Raji B cells were loaded with 5 μg/ml staphylococcal enterotoxin B for 45 min, and in parallel, PBTs were left untreated or treated with 100 μm or 1 mm 2-mercaptoethanol (β-ME). After washing out the staphylococcal enterotoxin B, PBTs and Raji cells were co-incubated to form contacts at a ratio of 1:1 for 45 min. For live/dead analysis, a sample was taken from each treatment, stained with 7-aminoactinomycin D (BD Biosciences) for 20 min at room temperature, and subjected to flow cytometry. The rest of each sample was successively fixed with 1.5% paraformaldehyde and permeabilized with 0.1% saponin. Cells were stained for F-actin (Alexa Fluor 488-conjugated phalloidin), cofilin (antiserum produced in our laboratory), CD3 (phycoerythrin/Texas Red-conjugated anti-CD3 antibody, BD Biosciences), and DNA (Hoechst 33342, Hoechst GmbH). After washing, the cells were centrifuged and resuspended in permeabilization buffer with phycoerythrin-coupled donkey anti-rabbit antibody to label anti-cofilin antibody. After removing the unbound antibody by two washing steps with Dulbecco's PBS, 24,000 cells/doublets were acquired in an Amnis IS-100 imaging flow cytometer. F-actin enrichment in the immune synapse of the T cells was evaluated by calculating the mean fluorescence intensity of T cell phalloidin staining in the area between each CD3^+^ cell-CD3^−^ cell doublet. From this, the percentage of doublets with high (top 50%), intermediate, and low (lowest 25%) enrichment of F-actin in the synapse was calculated.

##### Western Blot Analysis of the Cofilin Phosphorylation State

PBTs (1 × 10^6^/well) were incubated for 30 min in a 24-well plate in RPMI 1640 medium and 10% FCS containing 50 μm to 1 mm β-ME, 1–10 mm DTT, or no reducing agent. As a positive control, cofilin dephosphorylation was induced via T cell costimulation or via phorbol 12-myristate 13-acetate. To this end, either wells were coated with anti-CD3 antibody (20 ng/ml; clone OKT3, ATCC CRL-8001) or anti-CD28 antibody (5 μg/ml; BD Biosciences 555725), or 10 ng/ml phorbol 12-myristate 13-acetate was added to the cells. To exclude unspecific activation in the case of T cell costimulation, for control cells (IgG), wells were coated with IgG2aκ (20 ng/ml; BD Biosciences 553454) and IgG1κ (5 μg/ml; BD Biosciences 557273). After treatment with reducing agents, cells were lysed, and post-nuclear lysates were subjected to SDS-PAGE and Western blotting. Western blot membranes were probed for serine 3-phosphorylated cofilin (anti-phospho-cofilin Ser-3 antibody, Cell Signaling 3311L) and for total cofilin. The cofilin phosphorylation index for each sample was determined by first calculating the phospho-cofilin:cofilin ratio and then normalizing this to the ratio in untreated resting cells.

##### Flow Cytometry Conjugate Assay

Analysis of conjugate formation was performed with the samples obtained by imaging flow cytometry (see above). One additional surface staining with AmCyan-coupled anti-CD19 antibody (BD Biosciences) was performed in FACS wash (Dulbecco's PBS + 5% (v/v) FCS, 5% (w/v) BSA, and 0.07% (w/v) NaN_3_). Cells (10,000/sample) were acquired with a BD LSR II flow cytometer and analyzed for CD3 and CD19 staining.

##### Statistical Analysis

Data were statistically analyzed with GraphPad Prism 6.0a software by a two-tailed paired *t* test.

## RESULTS

### 

#### 

##### Cofilin Reduction Leads to Structural Changes

**FIGURE 1.**
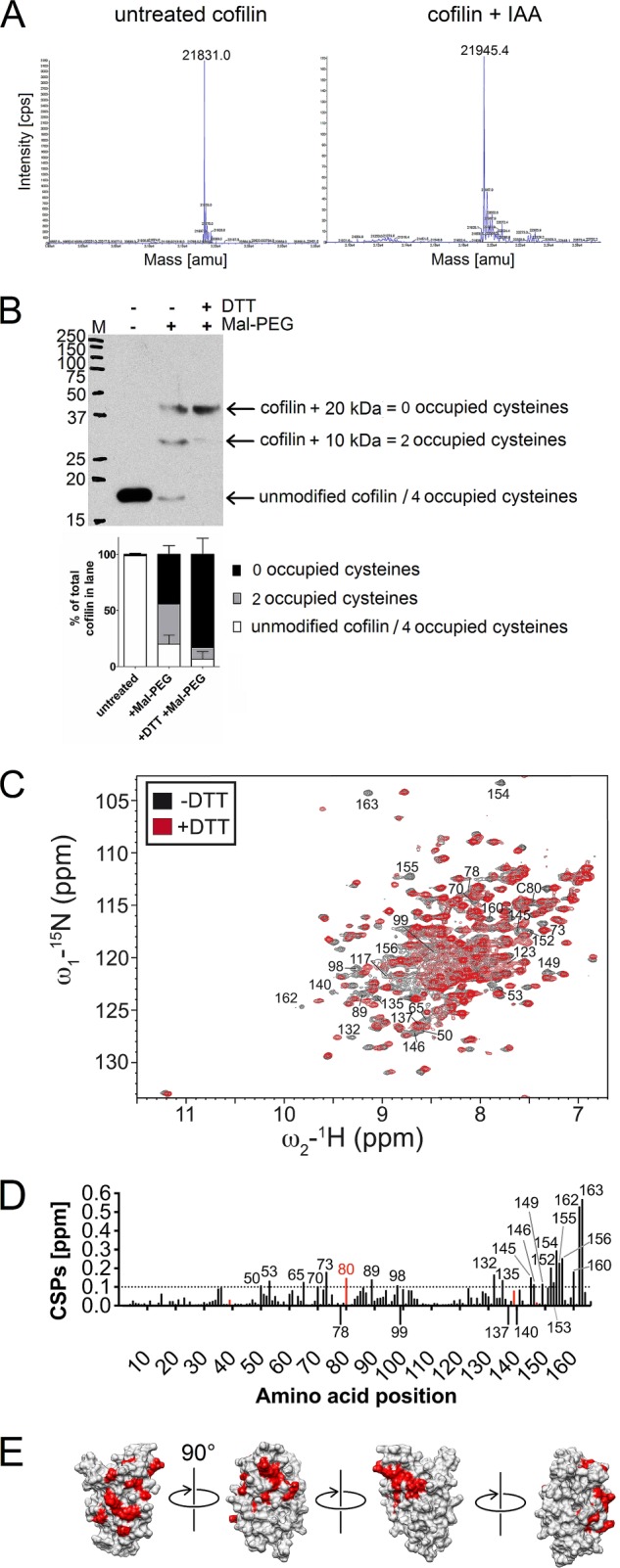
**Analysis of the cofilin redox state and its influence on cofilin structure.** *A*, mass spectrometry analysis of recombinant WT cofilin purified under nonreducing conditions. The His-tagged untreated molecule (*left spectrum*) shows a main mass peak at 21,831 atomic mass units (*amu*). Treatment with IAA (*right spectrum*) resulted in an increase in size by 114 atomic mass units (to 21,945 atomic mass units), corresponding to the addition of two IAA molecules. The accuracy of the spectrometer was ±1 atomic mass unit. *B*, Western blot of Mal-PEG cysteine modification assay performed with PBT lysate with and without reducing treatment. The membrane was probed for total cofilin; note that although the same amount of lysate was loaded on each lane, antibody detection decreased in Mal-PEGylated samples, probably due to epitope masking by Mal-PEG molecules. *M*, molecular mass markers shown in kilodaltons. *C*, the ^15^N-^1^H heteronuclear single quantum coherence spectrum of untreated cofilin (*black*) is overlaid with that of DTT-treated cofilin (*red*). Amino acids that exhibit distinct CSPs (>0.1 ppm) are labeled with their amino acid position. *D*, all CSPs occurring upon cofilin reduction. Again, all CSPs > 0.1 ppm are labeled; additionally, the four cysteines are marked in *red*. CSPs involving the complete disappearance of an amino acid peak in one of the spectra are marked by a *negative bar. E*, a three-dimensional rendering of the molecular surface of cofilin (PDB ID 1Q8G), with CSPs from the WT with/without DTT comparison colored *red*. Four views from around the molecule are shown by a perspective shift of 90° from one image to the next.

Cofilin contains four cysteines (at positions 39, 80, 139, and 147) and has been reported to be highly sensitive to oxidation and to form an intramolecular disulfide bridge under oxidative conditions. However, these observations were made on the basis of recombinant cofilin purified under reducing conditions. Therefore, here, we ask whether cofilin in its native state, purified under nonreducing conditions, already exists in an oxidized state. Thus, we analyzed the tagging of free cysteines with the alkylating agent IAA by mass spectrometry. IAA-treated recombinant cofilin showed an increase in mass of 114.4 (±1) atomic mass units (Fig. 1*A*), corresponding to the addition of two IAA molecules to cysteine side chains. This implies that two cysteines in cofilin are occupied and therefore could take part in a disulfide bridge.

We next asked whether the molecule in resting T cells also has two of its cysteines occupied. To investigate this cysteine modification, assays were performed on human PBT lysates with the alkylation agent Mal-PEG, which binds free thiols covalently and increases the size of the protein by 5 kDa per Mal-PEG addition. Two major forms of cofilin were detected in untreated PBT lysate (Fig. 1*B*): one form shifted upwards in size by two Mal-PEG molecules (10 kDa), and the other form shifted by four Mal-PEG molecules (20 kDa), corresponding to two and four free cysteines, respectively. There was also a minor fraction of cofilin that either was unmodified or had no free cysteines. To find also determine whether the occupied cysteines can be freed by a reducing agent, a lysate was DTT-treated and then Mal-PEGylated. A change in the Mal-PEGylation pattern toward more Mal-PEGylation would strongly indicate disulfide bridge formation in the protein. Indeed, upon reduction, only the cofilin with four free cysteines was detected (four Mal-PEG additions).

These results elicited the question as to whether structural changes occur in the cofilin molecule under different redox conditions and of what significance they are to the its function. Therefore, we recorded ^15^N heteronuclear single quantum coherence spectra of purified human cofilin before and after treatment with either the oxidant hydrogen peroxide or the reducing agent DTT. First, we determined that the WT cofilin + DTT spectrum recorded in this work is in very good accordance with the heteronuclear single quantum coherence peaks deposited in the Biological Magnetic Resonance Bank (BMRB) file (20) for human cofilin (BMRB entry 6004; reduced cofilin) (supplemental Fig. S1). The spectra measured after low oxidative treatment of cofilin showed no changes compared with the reduced WT spectrum (100 μm H_2_O_2_ treatment) (supplemental Fig. S2, *green*) but exhibited aggregation with a higher oxidant concentration (10 mm H_2_O_2_) (supplemental Fig. S2, *red*). In contrast, Fig. 1*C* shows that WT cofilin purified under nonreducing conditions underwent chemical shift changes when treated with DTT, which occurred in different regions of the molecule (Fig. 1*D*).

When the changes are marked on the three-dimensional structure of human cofilin (PDB 1Q8G), it becomes apparent that the amino acids exhibiting CSPs caused by reduction lie mainly in three clusters (shown in *red* in Fig. 1*E*, *first* through *third panels*) and some scattered amino acids near these clusters, whereas one side of the protein remains unaffected (*third* and *fourth* panels). All in all, 25 of 166 (15%) amino acids were affected by reduction.

As changes in the cofilin spectrum could be introduced into the protein by a reducing agent, they were likely to be caused by the breakup of disulfide bridges. This assumption is in line with the modification analyses of recombinant cofilin and cofilin from T cell lysates shown in Fig. 1.

##### Cofilin Cysteine Mutants Structurally Mimic the Reduced WT Protein

We purified four ^15^N-labeled mutants of the protein with each of the cysteines replaced with alanine (C39A, C80A, C139A, and C147A). Cysteine-to-alanine mutations were chosen because serine substitutions were unsuitable considering the importance of phosphorylation for cofilin activity (4), and alanine (after serine) is most similar to cysteine in side chain characteristics. If a cysteine mutant underwent the same structural changes upon reduction as the WT, it could be deduced that this particular cysteine was not part of a disulfide bridge of structural importance in the WT. We observed that, in contrast to C39A, C139A, and C147A, the C80A mutant was not properly folded after nonreducing purification (two separate expressions and purifications produced the same results). Therefore, C80A could not be analyzed in this comparison. Interestingly, when comparing the spectra of each of the other three mutants (C39A, C139A, and C147A) before and after reduction, none of them showed any significant CSPs caused by reduction (supplemental Fig. S3 contains overlays of the nonreduced form of each mutant with the reduced one).

**FIGURE 2.**
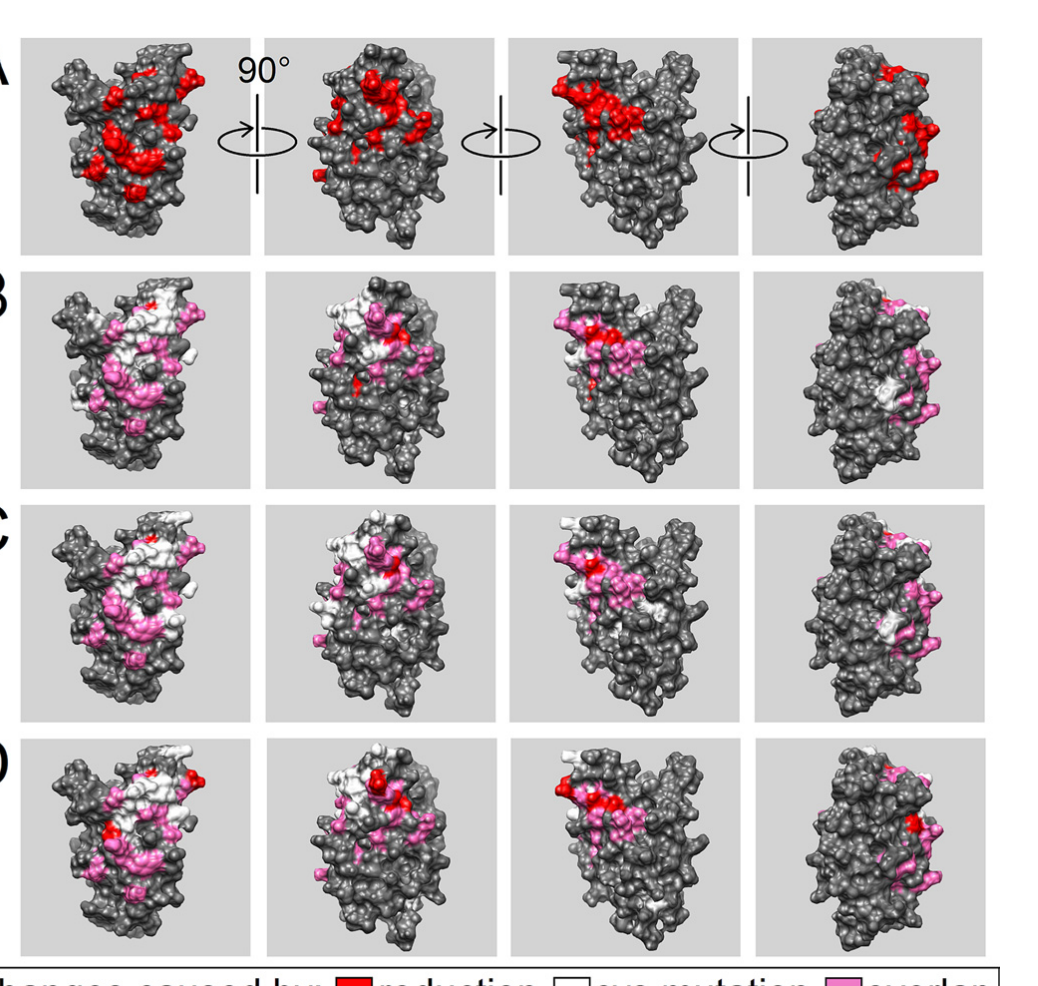
**CSP comparison of cofilin variants.** *A*, changes in the WT cofilin structure caused by reduction (*red*) superimposed on the three-dimensional surface model of the protein (PDB ID 1Q8G), as in Fig. 1*C. B*, overlay of CSPs > 0.1 ppm between mutant C39A and the nonreduced WT (*white*) and the changes shown in *A* (*red*). The overlap of both is colored *pink. C*, comparison of mutant C139A and nonreduced WT cofilin with the same colors as in *B. D*, comparison of mutant C147A and nonreduced WT cofilin with the same colors as in *B*.

If comparing the changes in WT cofilin caused by reduction (Fig. 1) with the differences between the WT and the three mutants, there is a clear disparity depending on which WT cofilin spectrum (with or without DTT) we used as the basis for comparison. Compared with the reduced WT, all three mutants have very low CSPs and therefore are very similar to the reduced WT (see supplemental Fig. S3 for spectra overlays and supplemental Fig. S4*A* for all CSPs from these overlays). However, from the direct comparison of NMR spectra with the nonreduced WT, it becomes apparent that a very similar pattern of change was triggered by either reduction of the WT or exchange of any single cysteine for alanine (see supplemental Fig. S4*B* for all CSPs from these comparisons and Fig. 2 for only CSPs > 0.1 ppm marked on the three-dimensional cofilin model). Amino acids 50, 53, 65, 73, 78, 80, 89, 99, 132, 135, 140, 145–146, 152, 154–155, and 162–163 show common chemical shift changes for the different cofilin forms (these overlaps are the amino acids shown in *pink* in Fig. 2, *B–D*).

Regarding the structural changes in WT cofilin caused by reduction, rather than showing a clear shift perturbation of two cysteines, which would allow the attribution of disulfide bridges to certain cysteines, only cysteine 80 was strongly affected by reduction of the WT. Mutating cysteines 39, 139, and 147 also affected cysteine 80, but each had no effect on the remaining two cysteines (supplemental Table S1).

##### Reduction of Cofilin Does Not Influence Its Actin-depolymerizing Activity

**FIGURE 3.**
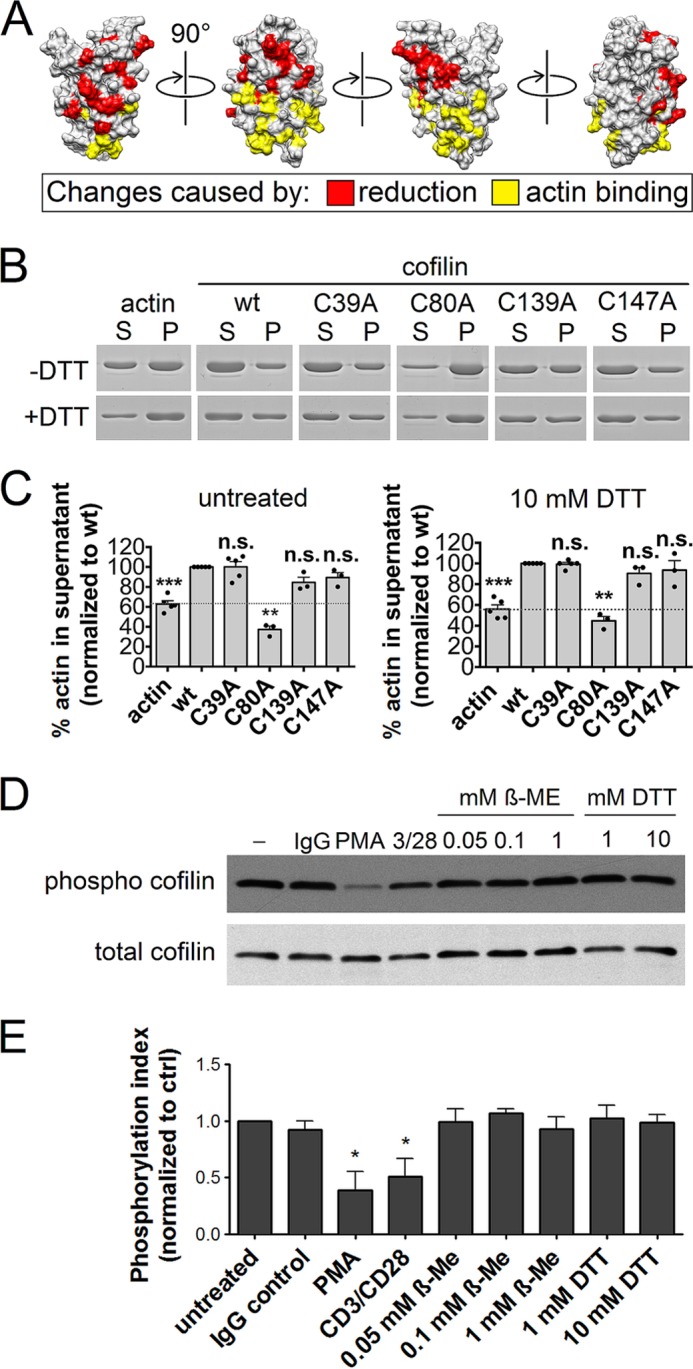
**F-actin-depolymerizing capacity of cofilin variants under nonreducing and reducing conditions.** *A*, the changes caused in cofilin by reduction are compared with the changes caused by actin binding to cofilin (summarized by Gorbatyuk *et al.* (24)). Amino acids influenced by actin binding are shown in *yellow*, and changes caused by reduction are shown in *red*, as in Fig. 1*C. B*, F-actin depolymerization assay of WT cofilin and the cysteine mutants showed the independence of F-actin-depolymerizing activity from DTT treatment. *S*, supernatant fraction after sedimentation (ultracentrifugation); *P*, pellet fraction after sedimentation. All bands shown are from one and the same experiment and partly from the same SDS gel; for clarity, *white dividers* have been put between each fraction couple. *C*, statistical evaluation of the F-actin-depolymerizing capacity of cofilin variants. ***, *p* < 0.001 compared with the WT (Student's two-tailed paired *t* test); **, *p* < 0.01 (*n* ≥ 3); *n.s.*, not significant. *D*, Western blot analysis of the cofilin phosphorylation in human PBTs in reducing milieus. Phorbol 12-myristate 13-acetate (*PMA*; soluble inducer) and CD3/CD28 stimulation of cofilin dephosphorylation are shown as positive controls. This Western blot is representative of three independent experiments. *E*, quantification of the cofilin phosphorylation state. *, *p* < 0.05 (Student's two-tailed paired *t* test). *ctrl*, control.

Having discovered the structural changes that can be introduced into cofilin by reduction or cysteine mutation, we questioned what function this change might have. The amino acids affected by DTT did not overlap at all with the actin-binding sites (shown in *yellow* in Fig. 3*A*) revealed by earlier studies (summarized in Ref. 24).

Notably, residues reported to be crucial for actin binding, namely lysine 96 and the phosphorylation site serine 3 (20), were not influenced by reduction. Nevertheless, in many areas of the protein, the changes caused by actin binding lie right next to residues affected by reduction. Therefore, we first tested whether untreated and reduced WT cofilin forms are equally capable of performing their function, *i.e.* depolymerizing F-actin. This was done via an actin depolymerization assay, and both nonreduced and reduced WT cofilin forms were able to depolymerize F-actin (Fig. 3, *B*, *second panels*, and *C*, *left* and *right panels*, *second bars*). Note that no spontaneous actin depolymerization occurred in the presence of DTT (supplemental Fig. S5). Furthermore, one of the most important cofilin regulatory mechanisms is the inhibitory phosphorylation on serine 3. Thus, we next determined the cofilin phosphorylation state in PBTs via Western blotting after incubation of live PBTs with different concentrations of β-ME or DTT. Stimulation with a soluble agent (phorbol 12-myristate 13-acetate) or with immobilized antibodies (anti-CD3/CD28) led to the expected dephosphorylation of cofilin (Fig. 3, *D* and *E*). In contrast yet consistent with the analysis of cofilin activity under reducing conditions, the phosphorylation status of T cell cofilin was not significantly altered by different reducing treatments (Fig. 3, *D* and *E*).

To test whether the cysteine mutants of cofilin are as active as the WT, they were subjected to the same assay. The results show that mutants C39A, C139A, and C147A harbor a strong actin-depolymerizing capacity with and without DTT (Fig. 3, *B* and *C*). However, mutating the surface cysteines 139 and 147 led to slightly less activity than the WT under nonreducing conditions, but this was not statistically significant. In contrast, mutant C80A completely lost its actin-depolymerizing activity (Fig. 3*B*, *left* and *right panels*, *fourth bars*). This was to be expected because the mutant was found to be unfolded in NMR analysis.

##### Reducing Treatment Rescues Cofilin from PIP_2_ Inhibition

**FIGURE 4.**
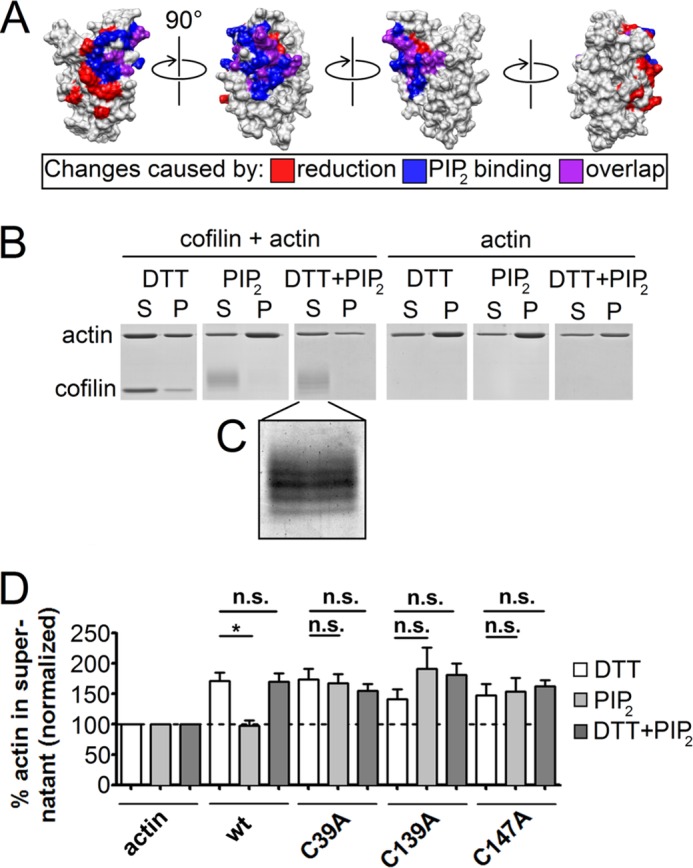
**Analysis of the actin-depolymerizing capacity of reduced WT cofilin and cysteine mutants in the presence of PIP_2_.** *A*, cofilin residues affected by PIP_2_ binding (as reported by Gorbatyuk *et al.* (24)) are shown in *blue*, changes upon reduction are shown in *red*, and changes caused by both PIP_2_ binding and DTT are shown in *purple. B*, F-actin-depolymerizing capacity of WT cofilin under nonreducing/reducing conditions and in the presence or absence of PIP_2_. The treatment combinations are shown above the lanes. *Left panels*, pretreated cofilin and actin; *right panels*, actin alone underwent the corresponding treatments and the assay. *S*, supernatant; *P*, pellet of the depolymerization assay. *White dividers* were inserted for clarity. *C*, a magnified detail from the gel indicated displays the upward shifts of cofilin following binding to PIP_2_ (1 kDa per PIP_2_ molecule). The image was enhanced by optimizing contrast for better visibility. *D*, results of a paired *t* test from actin sedimentation assay of WT cofilin and cysteine mutants with PIP_2_. Statistical significances (*, *p* < 0.05) were calculated for each cofilin form with PIP and with DTT and PIP_2_ compared with the same form with DTT after each had been normalized to the corresponding actin control, which underwent the same treatment (*n* ≥ 3). *n.s.*, not significant.

After ensuring that the F-actin-depolymerizing function of cofilin was not influenced by reduction, we considered that the observed structural changes might alter cofilin interaction with other binding partners. Interestingly, we found that the changes caused by reduction overlapped with the PIP_2_-binding site of cofilin (mapped by Gorbatyuk *et al.* (24)). The amino acids affected by reduction partly match the cofilin residues influenced by PIP_2_ binding (Fig. 4*A*, shown in *purple*) and partly lie adjacent to them (Fig. 4*A*, shown in *blue*).

Accordingly, we asked if these changes (also mimicked by mutants C39A, C139A, and C147A) have an impact on PIP_2_ regulation of cofilin function. We tested untreated and reduced WT cofilin forms in actin sedimentation assays in both the presence and absence of PIP_2_. Notably, although the actin-depolymerizing activity of untreated cofilin was completely suppressed by PIP_2_, reduced WT cofilin was rescued from PIP_2_ inhibition (Fig. 4*B* and *D*). To further corroborate our findings on the similar structural changes between the mutants and WT cofilin, all three functional cysteine mutants were tested for actin-depolymerizing activity in the presence and absence of PIP_2_ and indeed exhibited the same escape from PIP_2_ inhibition as reduced WT cofilin (Fig. 4*D*). Note that the presence or absence of DTT in addition to PIP_2_ made no significant difference in the depolymerizing activity of the mutants. Moreover, this assay allowed us to verify that PIP_2_ was still binding to all the untreated and DTT-treated cofilin forms, as cofilin shifted upwards in the gel by the expected size (1092 Da) or a multiple thereof only if it had been incubated with PIP_2_ (see Fig. 4*B* (*left panels*) for a representative example). Fig. 4*C* shows a magnified view of the cofilin upwards shifts in the gel. Note that the image was contrast-enhanced to increase visibility.

##### A Reducing Micromilieu Lowers the F-actin Enrichment in the Immune Synapse

**FIGURE 5.**
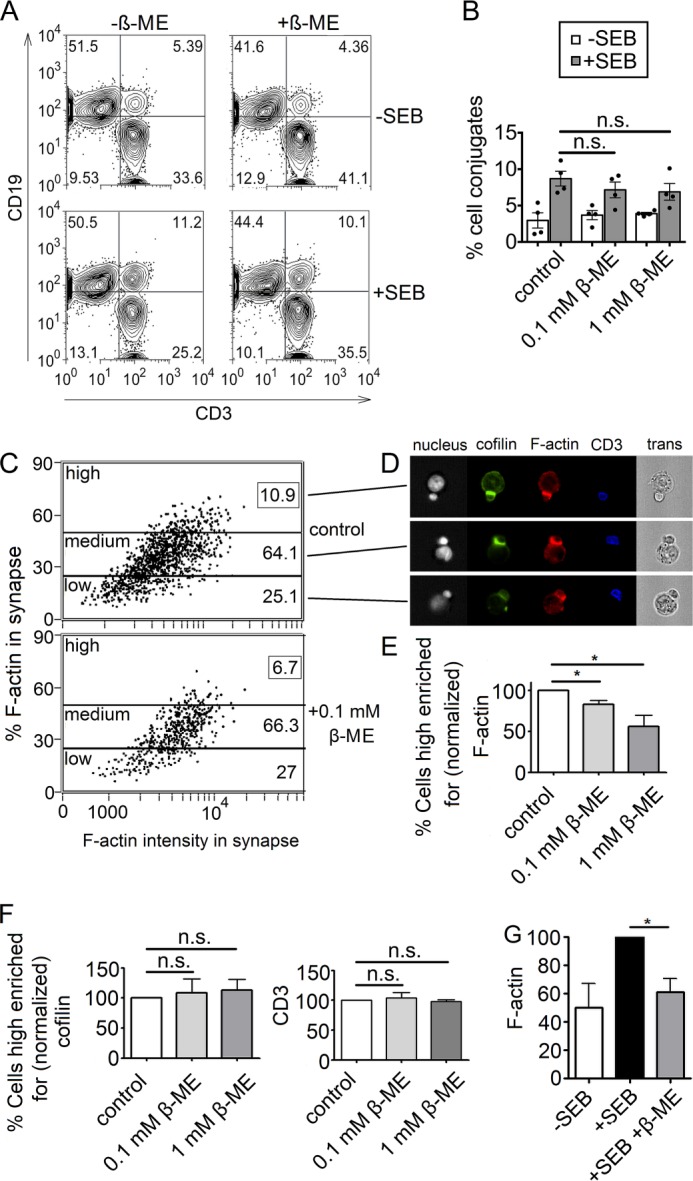
**Analysis of PBT-APC contacts for F-actin enrichment in the immune synapse after reducing treatment.** *A*, flow cytometry analysis of T cell-APC conjugates formed with untreated or 1 mm β-ME-treated PBTs. The extent of contact formation was determined by the percentage of CD3/CD19 double-positive events. *B*, quantification of conjugation assay after β-ME treatment. The data were statistically analyzed with a two-tailed paired *t* test (*n* = 3). *C*, cell contacts imaged by multispectral imaging flow cytometry divided into contacts with high, medium, and low F-actin enrichment in the T cell part of the synapse. *Numbers* in the gate designate the percentage of cells in the entire population of contacts. *D*, exemplary in-flow microscopy images of T cell-APC contacts with high (*upper panel*), medium (*middle panel*), or low (*lower panel*) F-actin-enriched T cells. *trans*, transmitted light microscopic image. *E*, quantification of T cells with high F-actin enrichment in the contact zone. *F*, quantification of T cells with high cofilin (*left panel*) or CD3 (*right panel*). *G*, comparison of the F-actin enrichment between contacts in the absence or presence of superantigen (staphylococcal enterotoxin B (*SEB*)). *, *p* < 0.05 in a two-tailed paired *t* test (*n* = 3); *n.s.*, not significant.

Having established the fact that PIP_2_ inhibition of cofilin is prevented by reduction of cofilin, we investigated what effect a reducing milieu might have on the actin cytoskeleton during T cell activation. Given that even PIP_2_-bound cofilin is able to depolymerize F-actin under reducing conditions, we reasoned that this might lead to decreased F-actin content by increased actin dynamics in the immune synapse between T cells and APCs. Specifically, to test for possible consequences of cofilin reduction on the function of primary human T cells, we examined the effect of β-ME on F-actin enrichment in the T cell-APC immune synapse, where PIP_2_, as part of the plasma membrane, and cofilin, accumulating in the distal and peripheral supramolecular activation complexes, colocalize. After we excluded an influence of the reducing agent on the number of cell contacts forming (Fig. 5, *A* and *B*) and on cell viability (supplemental Fig. S6), we examined F-actin and cofilin enrichment in the contact zone between T cells and staphylococcal enterotoxin B-loaded antigen-presenting B cells.

As a result of T cell pretreatment with β-ME, the fraction of cells forming immune synapses with high F-actin enrichment was significantly reduced (Fig. 5, *C–E*), even though equal amounts of cofilin and CD3 were enriched in these synapses (Fig. 5*F*). It could also be confirmed that β-ME increased actin dynamics, so the F-actin amounts were comparable to those observed in contacts formed in the absence of superantigen (Fig. 5*G*). This fits nicely with our previous *in vitro* findings that the actin-depolymerizing activity of cofilin in the presence of PIP_2_ can be reconstituted in a reducing milieu.

## DISCUSSION

We have shown for the first time that cofilin structure and regulation are altered by a reducing milieu. So far, many mechanisms of the oxidative regulation of T cell signaling have been described. The opposite process, that of producing the more reduced forms of proteins, has been largely neglected, even though many circumstances have been described in which a reducing milieu has a strong effect on the function, survival, and behavior of cells (17–19). We identified the amino acids of cofilin involved in structural changes between the nonreduced and reduced molecules, and we revealed a common pattern of amino acids influenced both by cysteine mutations and by reduction of cofilin. From these observed changes, we deduced and proved the impact on PIP_2_ regulation of cofilin: reduced WT cofilin and the cofilin cysteine mutants were not inhibited by PIP_2_ binding.

Interestingly, if cysteine 39, 139, or 147 was replaced, the NMR spectra overlay with the reduced WT revealed overall very low CSPs (except for a few distinct regions, but these were mostly in the vicinity of the mutated residues), whereas all three mutants had a similar pattern of CSPs compared with the nonreduced WT. This suggests that, by any of the manipulations, a very similar structure emerges, which led to the hypothesis that mutants C39A, C139A, and C147A mimic the reduced cofilin structurally. These results, unexpected as they were because of the different positions and environments of the four cysteines, led us to the conclusion that all cysteines in cofilin are crucial for the production of cofilin with an intramolecular disulfide bridge, most likely between cysteines 39 and 80. It cannot be excluded that, in this state, a second disulfide bridge exists in a small fraction of the protein, as in Mal-PEG modification assays, a minor fraction of unmodified protein was observed. Although cysteine 80 was revealed to be essential for any stable form of cofilin, replacement of the other three cysteines led to similar results structure-wise. We hypothesize from this observation that, during production of cofilin, all cysteines are crucial for a stepwise disulfide isomerization process. Such a process of self-chaperoning has already been described for proteins with homology to actin-remodeling factors (25). From the changes in the cofilin NMR spectrum caused by reduction, we hypothesize that cofilin in its natural condition after expression exists in a redox state different from the reduced state of the protein, *i.e.* it most likely has at least one disulfide bridge responsible both for the structural conformation described here and for its gel shift behavior upon nonreducing SDS-PAGE described in one of our previous studies (14). As to the exact location of a possible disulfide bridge, the most likely cysteines participating in such a bond are at positions 39 and 80, as they are in close proximity in the three-dimensional structure, whereas cysteines 139 and 147 are much further apart and also remote from both cysteines 39 and 80, respectively. The presence of this disulfide bridge might not be obvious by strong changes in both participating cysteines in NMR analysis if only one of the partnering cysteines moved toward the other to form the bond. Again, cysteines 39 and 80 are at such a distance from each other to enable the formation of a disulfide bridge (11.01 Å in the reduced structure, measured between the sulfur atoms of the cysteines) (supplemental Fig. S7). In addition, a molecular dynamics simulation of cofilin PDB structure 1Q8G under the premise of a disulfide bridge between cysteines 39 and 80 yielded root mean square deviations for the sulfur atoms of cysteines 39 and 80 of 3.44 and 6.59, respectively (the maximum root mean square deviation in this simulation was 8.26). Thus, although both participating cysteines would be altered in their position in the protein, cysteine 80 would be among the amino acids most affected by the presumed disulfide, whereas the effect of the disulfide bond on cysteine 39 was less pronounced. In this simulation, cysteine 80 must rearrange to a much stronger degree than cysteine 39 to bond, consistent with the changes we observed in WT cofilin NMR spectra before and after reduction.

In one of our earlier studies (14), we analyzed the activity and redox state of cofilin under oxidative stress and speculated on the formation of a disulfide bridge in the molecule under oxidizing conditions. However, it could not be elucidated at the time if the original folding of untreated cofilin already included a disulfide bridge. Our new data expand the picture of the different cofilin states in that T cell cofilin exists in two major distinct pools side by side, with one form completely reduced and the other with one disulfide bridge. In addition, a third minor form with two disulfide bridges could exist in T cells. In our model, a reducing milieu can tip the balance in the direction of cofilin without any disulfide bridges.

On the other hand, at higher oxidant levels, an aggregation of cofilin takes place, likely caused by the formation of intermolecular disulfide bridges and thus a multimerization among cofilin molecules. The existence of intermolecularly connected cofilin oligomers under oxidative stress has already been described by others (27–29); these oligomeric protein forms might protect proteins from the formation of more highly oxidized forms (26). We could trace this back to the two surface cysteines: the disulfide formation between protein monomers would most likely occur via their surface cysteines, *i.e.* in the case of cofilin cysteines 139 and 147. Therefore, removing these cysteines would prevent intermolecular connections. Indeed, a C139A/C147A double mutant of the protein proved to be much more stable than the WT against oxidative stress (compare supplemental Figs. S2 and S8). On the other hand, the structural changes resulting from reducing conditions revealed in this work are very likely caused by the breakup of an intramolecular disulfide bridge upon reduction because neither nonreduced nor reduced WT cofilin exhibited any aggregation. On the contrary, the observed structural change is taking place in each individual molecule by itself, as intermolecular interaction would register in the NMR spectrum.

The information from the cysteine modification assay, taken together with the higher resistance against oxidation of the C139A/C147A double mutant, leads us to the conclusion that a disulfide bridge exists in the native molecule between cysteines 39 and 80, which is broken up in a reducing milieu. In contrast, the outer cysteines can participate in intermolecular disulfide bridge formation in an oxidizing environment.

In light of the new structural information obtained from NMR and Mal-PEGylation assays regarding the reduction of WT cofilin, we proceeded with functional testing of WT cofilin and the cysteine mutants under nonreducing and reducing conditions. The assessment of cofilin activity in the presence and absence of DTT showed that neither the F-actin-depolymerizing function of WT cofilin nor that of mutants C39A, C139A, and C147A changed with reduction. Therefore, we conclude that F-actin binding and depolymerization are not hindered by the structural changes described above. When tested in the presence of PIP_2_, the F-actin-depolymerizing activity of WT cofilin was suppressed, as has been described previously (9). Interestingly, the reduction of WT cofilin changed this situation drastically in that PIP_2_ still bound to cofilin but was unable to inhibit it. Mutations of cysteines 39, 139, and 147 had the same effect. This fits nicely with the fact that the mutants did not undergo structural changes upon reduction but had already adopted a conformation similar to that of the reduced WT. From this we conclude that mutants C39A, C139A, and C147A mimic the reduced form of WT cofilin with regard to PIP_2_ regulation.

We next asked what effect a reducing micromilieu would have on the actin cytoskeleton of living T cells undergoing activation by APCs. We saw a dose-dependent reduction in the percentage of T cells with high F-actin enrichment in the immune synapse with increasing concentrations of reducing agent. This is in good accordance with the conjecture drawn from the earlier functional data presented here, namely that in a reducing micromilieu, the cofilin pool bound by PIP_2_ becomes active. With quantitatively more active cofilin available in the immune synapse, the actin cytoskeleton would become more dynamic by increased F-actin depolymerization and severing. Such a change in the amount of active cofilin in the immune synapse can have a high impact on the affected T cell because, first, this modulation of cofilin activity is of a spatially confined nature, as PIP_2_ is anchored in the plasma membrane. Second, the transfer of the reducing milieu from dendritic cells to T cells was shown to be cell-specific in that only antigen-specific T cells profit from it, whereas T cells specific for an unrelated antigen remained unprotected against oxidative stress (17). The functional consequences of cofilin reduction in PBTs are currently under investigation. Regarding our experiments with living cells, we cannot at this point exclude that other mechanisms of β-ME action affect the outcome we describe here. A multifactorial cause for the observed change in F-actin enrichment is likely in fact because, for example, gelsolin, another actin-binding protein that has F-actin-severing activity similar to that of cofilin, is known to be redox-sensitive as well (30).

Studies on T cell activation and the formation of the immune synapse have revealed an intricate network of competing pathways toward cofilin regulation: both the inactivating pathway (via LIM kinase) and two activating pathways (via PI3K and via phospholipase C) take part in the signaling downstream of costimulation (31–33). At present, we do not know of any model explaining the co-occurrence and interplay of these pathways during T cell costimulation. In tumor cells, simultaneous phospholipase C and LIM kinase activities were shown to lead to the spatial and temporal control over cofilin activity necessary for proper chemotaxis (12, 34). A spatial compartmentalization of cofilin regulation arises from the fact that, as long as PIP_2_ binds cofilin, it is restricted to the membrane. In carcinoma cells, a pool of cofilin bound to PIP_2_ was observed (35). This cofilin was inactive, as it was inhibited by PIP_2_, but it was noted that all of the cofilin in this pool was dephosphorylated, *i.e.* not inhibited by phosphorylation (36). Regarding the hydrolysis of PIP_2_ triggered by costimulation, it is essential to keep in mind that 40–60% of the initial PIP_2_ remains uncleaved at the plasma membrane throughout the process of cell activation, harboring an inactive cofilin pool (12), which in turn clusters PIP_2_ molecules together (35). Upon PIP_2_ hydrolysis, active cofilin is freed locally at the membrane to reorganize cortical actin. If, however, cofilin diffuses away into the cytosol, it becomes phosphorylated by LIM kinase. This division in space of active and inactive cofilin populations inside one cell was concluded to render asymmetrical movements such as directed migration possible. The fact that a reducing milieu does not influence the phosphorylation state of cofilin in T cells underlines the localized effect of cofilin reduction further because reduction of unbound cofilin in the cytosol does not affect its activity. From our data, we speculate that reduction of cofilin allows for continued cofilin activity while anchoring it to the membrane through PIP_2_. It has hitherto been described that the different regulatory mechanisms (phosphorylation, PIP_2_ binding) affecting cofilin work in a redundant fashion. Although we could demonstrate that cofilin reduction has no direct effect upon its phosphorylation status, with the mechanism we identified here we present another possible regulatory layer to modulation of the actin cytoskeleton as it occurs, for example, during immune synapse formation and T cell activation: in a reducing milieu, a spatially confined pool of otherwise inhibited cofilin becomes active and contributes to actin cytoskeleton rearrangement near the membrane. This mechanism may explain why dendritic cells that are able to increase the thiol pool in antigen-specific T cells enable T cell activation even under oxidative stress conditions (17–19).

## Footnotes

4 The abbreviations used are:

PIP_2_: phosphatidylinositol 4,5-bisphosphate
IAA: iodoacetamide
PDB: Protein Data Bank
CSP: chemical shift perturbation
PBT: peripheral blood T cell
Mal-PEG: methoxypolyethylene glycol maleimide
APC: antigen-presenting cell
β-ME: 2-mercaptoethanol.
